# Socio-Psychological Factors Associated with Young Australian Adults’ Consumption of Energy Dense and Nutrient Poor (EDNP) Foods

**DOI:** 10.3390/nu14040812

**Published:** 2022-02-15

**Authors:** Krupa Thammaiah Kombanda, Claire Margerison, Alison Booth, Anthony Worsley

**Affiliations:** Institute for Physical Activity and Nutrition (IPAN), School of Exercise and Nutrition Sciences, Deakin University, Geelong, VIC 3220, Australia; claire.margerison@deakin.edu.au (C.M.); alison.booth@deakin.edu.au (A.B.); anthony.worsley@deakin.edu.au (A.W.)

**Keywords:** young adults, Australia, qualitative, food practices, socio-psychological factors, Energy Dense and Nutrient Poor foods, unhealthy food choices, discretionary foods

## Abstract

Young Australian adults’ exhibit high consumption of Energy Dense and Nutrient Poor (EDNP) foods; however, there is limited research concerning the factors influencing their consumption. This study aimed to explore socio-psychological factors associated with young Australian adults’ (18–30 years) consumption of EDNP foods with consideration of the Food Related Lifestyle Model (FRLM) as a potential framework. Through qualitative descriptive research methodology, 38 young adults were interviewed. Data were thematically analyzed. Participants were classified into three groups based on their living arrangements namely, parental, shared and independent households. Five themes emerged, (1) psychological factors (2) intrinsic qualities of EDNP foods, (3) social factors, (4) accessibility and affordability and (5) health related beliefs. The FRLM takes into consideration some of the factors reported in this study as influencers of EDNP food intakes. However, the FRLM omits important psychological factors (motivation, restraint, cravings, coping strategies and habits) identified by participants as influencers over their EDNP food intakes. The FRLM may need to be extended in its application to EDNP food intakes of young Australian adults. Social marketing campaigns highlighting health risks, addressing social and environmental factors are suggested. The social desirability of healthier alternatives in social gatherings of young adults could be increased.

## 1. Introduction

Australia has one of the world’s highest prevalence of overweight and obese people [1]. In 2017–2018, nearly two-thirds of Australians aged 18 years and above (67%) were recorded as overweight (36%) or obese (31%) [2]. This equates to over 12.5 million Australian adults [2]. Globally most age cohorts (adolescents, young adults and adults have been gaining weight rapidly during the last few years; however, it is young adults (18–30 years) who have been showing the greatest weight gain in high-income countries such as Australia [3,4,5,6].

This is concerning as overweight and obesity increase the risk of developing Non-Communicable Diseases (NCD’s) such as type 2 diabetes mellitus, metabolic syndrome, cardiovascular diseases and certain cancers [7] which in turn increase the risk of premature death [8]. While there are several factors that increase NCD risk, the key factors leading to the burden of disease in Australia and elsewhere are unhealthy body mass index (overweight or obesity), poor intake of fruit and vegetables and a high intake of Energy Dense and Nutrient Poor (EDNP) foods or discretionary foods [9]. 

Limited consumption of fruit and vegetables and the excessive intake of EDNP foods are commonly observed behaviours among young Australian adults. The most recent Australian National Health Survey showed that young adults have the highest intake of sugar-sweetened beverages when compared to all adult age groups and over 35% of their total energy intake is attributable to EDNP foods [10]. Most importantly, only 4% of young Australian adults meet the recommended two fruit and five serves of vegetables per day [10]. Further, this group also exhibited relatively high intakes of foods prepared outside the home including take-away, restaurant and fast foods [11].

While it is clear that young Australian adults are a target audience for public health programs [8], little research has explicitly focussed on the discretionary or EDNP food intakes of young adults. A recent study in 2016 examined the factors influencing 8 to 14-year-old Australian children’s consumption of EDNP foods and noted parents’ permissive attitudes, children’s requests for discretionary foods and perceived social norms as influencing factors [12]. Moore et al. (2019) examined the EDNP food intakes of the general Australian adult population but not those of young adults specifically [13]. Therefore, this is an important research gap.

Furthermore, young adulthood is a transitional period between adolescence and adulthood and is associated with the development of self-identity, self-reliance and autonomy [14]. Many young adults leave home during this time for further education and/or employment and many involve themselves in important family formation behaviours including marriage, cohabitation and parenting [15]. Although past quantitative studies suggest that living arrangements play an important role in determining young adults’ food behaviours [16,17,18,19,20,21] during this transitional period, there is a lack of contextual and in-depth information concerning how or in what ways living arrangements influence young adults’ food behaviours. Additionally, these studies have focussed more on healthy eating behaviours than discretionary food behaviours. Therefore, understanding the potential influence of living arrangements may be crucial towards improving young adults’ food behaviours as they transition from dependent to independent living. This is a priority as the period of young adulthood lays foundation for long-term sustenance of healthy food behaviours [22]. 

Extensive research has focused on the barriers and facilitators of ‘healthy eating’ among young adults [18,23,24,25,26,27,28]. These barriers and facilitators include taste preferences, subjective norms, availability and affordability, nutrition knowledge, cooking skills, food-related planning, social support or encouragement, weight management, self-esteem, motivation and self-regulatory skills [26]. While these socio-psychological factors have been shown to be related to healthy eating, factors influencing the consumption of EDNP foods may differ and need to be investigated and defined. 

The Food Related Lifestyle Model (FRLM) proposed by Grunert et al. (1993) has been used in the past to examine consumers’ food related lifestyles. The FRLM suggests that consumption-related lifestyles are a set of cognitive categories and scripts that associate a set of food products with a set of values [29]. The FRLM consists of six main components namely, cooking scripts, shopping scripts, usage situations, desired higher-order product attributes or quality expectations, concrete attributes or product categories, and consequences [29]. Originally, the FRLM model was operationalised through a questionnaire with 69 items to measure five dimensions on a 7-point Likert scale [30]. The model has been used in many food studies to understand consumers’ perceptions including consumption of fruits [31], functional foods [32], ready to eat foods [33], organic foods [34] and across nations [32], demonstrating remarkable stability and reproducibility. However, the FRLM has not been employed to understand factors influencing young Australian adults’ consumption of EDNP foods. Moreover, the FRLM does not take into account socio-psychological factors such as the motivational constructs [35], habits [36], social norms and impulsivity. This provides an opportunity for further research.

Further, the Food Related Lifestyle Model includes additional features aside from those of the ‘Theory of Reasoned Action’ [37], and its extension, the ‘Theory of Planned Behaviour’ [38] which have been the most dominant social psychological models used for predicting the determinants of food choice [39]. A major advantage of the FRLM is its comparative simplicity given the complexity of the aetiology of food consumption [35]. Henceforth, the FRLM appears to be an appropriate framework to understand the socio-psychological factors influencing food consumption.

It is important to understand the influence of socio-psychological factors as past research has indicated that motivation, impulsivity, habits and social norms have been commonly associated with young adults’ food behaviours. However, these have not been examined in the Australian context. A lack of motivation, such as risk-taking behaviour, appears to be a barrier for healthy eating among young adults [26]. Impulsivity has been identified as a psychological factor that drives young adults’ health behaviours [40]. Social norms have also been identified as important influences over young adult’s food behaviours [41]. Habit strength has been an important predictor of adults’ food behaviours including those of unhealthy snacking [36,42,43]. 

This evidence suggests that beyond the FRLM, there could be other socio-psychological factors, which may influence young adults’ consumption of discretionary foods, and these must be further explored. In summary, this qualitative study aimed to explore the socio-psychological factors associated with young Australian adults’ consumption of EDNP foods using the FRLM as a template.

The terms ‘discretionary foods’ and ‘unhealthy food choices’ were used in this study to refer to all EDNP foods. The Australian Dietary Guidelines [44] refers to discretionary foods as EDNP foods. These are defined as ‘food and drinks not necessary to provide the nutrients the body needs, but they may add variety’ [44]. Examples include sweet biscuits (cookies), cakes, desserts and pastries, processed meats and fattier or salty sausages, sweetened condensed milk, confectionery and chocolates, to name a few [44]. 

## 2. Materials and Methods

Qualitative descriptive research methodology [45] was chosen to obtain a detailed understanding of young adults’ views of the factors that influence their consumption of discretionary foods. This study adopted interpretivism as its research paradigm. Interpretivism relies upon ‘participants’ perspectives of the situation being studied [46]. Subsequently, participants were asked broad questions so that they could develop their own interpretation of the circumstance such as the likely causes of their consumption of EDNP foods without being influenced by the researcher [47]. Participants’ perceptions in qualitative descriptive research enabled presentation of the underlying causes that result in discretionary food behaviours [48]. All subjects gave their informed consent for inclusion before they participated in the study. The study was conducted in accordance with the Declaration of Helsinki and approved by the Faculty of Health Ethics Advisory Group (HEAG-H 18_2020, date of approval-17 March 2020) of Deakin University.

### 2.1. Participants and Recruitment

Young adults aged 18–30 years were recruited Australia-wide by posting advertisements on Facebook and Twitter. All advertisements were posted between July-September 2020 on Deakin University’s official social media accounts and Deakin University’s Institute for Physical Activity and Nutrition (IPAN) website. Maximum variation sampling was the strategy employed to ensure heterogeneity [49]. Three key demographic factors were considered during recruitment, these included living arrangements, student status and employment status. This study outlined two inclusion criteria: participants had to be aged between 18 and 30 years and residing in Australia. Before every interview, written consent and permission for audio-recording were obtained from participants. Young adults were each offered a $25 shopping voucher as compensation for their time. The researchers did not hold any previous relationships with the participants. Final sample size was informed by data saturation [50]. At the 38th interview, no new themes or sub-themes were noted, and data saturation was achieved. 

### 2.2. Interview Procedure

Interested participants were asked to email the lead researcher. Consequently, the researcher confirmed the eligibility and scheduled an interview. All interviews were conducted online using Zoom/Skype or over the phone. Interviews lasted between 39 to 72 minutes with an average duration of 46 minutes, in entirety (17 questions). 

An interview guide with open-ended questions and probes was developed based on a review of the literature. To determine the face validity of the questions, four pre-test interviews were conducted prior to the main study. Minor modifications were made to three questions based on the suggestions. However, the four pre-test interviews were not included in the final analysis. This paper includes analysis of six broad questions that related specifically to socio-psychological factors with six follow-up probes (See Table 1) out of the 17 questions comprising the broader study examining food behaviours of young Australian adults. 

### 2.3. Data Analysis

Participants were categorised into three groups based on their living arrangements. The classification was based on previous research conducted by the Australian Housing and Urban Research Institute [51]. The three living arrangement groups included: (1), young adults living in the parental/multi-family home which included dependant as well as non-dependant young adults and some couples or single parents in multi-familial homes of origin (“parental households”); (2), young adults living with related or unrelated people with/without children which may include other families, however, not including parent (s) (“shared households”); and (3) living independently, either as a couple with/without children or living independently as a single person household (“independent households”) [51].

The lead researcher transcribed the first two interviews manually to familiarize herself with the data [52]. The qualitative data analysis software package, NVivo 12.0 Plus for Windows (QSR International 2018) was used for data analysis. Remaining interviews were transcribed verbatim by a professional transcription service (Rev.com, last accessed—30 November 2020). Data analysis followed an iterative process [53], commencing after the transcription of the first interview. Template analysis technique was employed for data analysis [54]. Template Analysis is a type of thematic analysis that includes hierarchical coding of qualitative data while demonstrating flexibility to adjust to the needs of a specific study [55]. Template analysis involves the creation of initial coding templates. In the case of the present study, data analysis began by the formulation of a priori codes based on the research questions [55]. The steps of the template analysis followed were, (1) becoming familiar with the data; (2) preliminary data coding that is applying the priori codes identified in advance, modifying and developing new codes on the subset of data (first six interviews); (3) organization of themes into clusters and defining their relationships; (4) defining initial coding templates; (5) application of initial coding templates to further data and modifying and (6) finalization of template and application to the remaining data [52]. 

To ensure rigour, reliability and validity, member checks (respondent validation), audio-recording, inter-coding agreement were performed. Subsequently, participants were provided with an opportunity to review their transcripts. Accordingly, transcripts were emailed to eight participants who expressed an interest. Five out of eight participants returned the transcript without suggesting any changes. Three did not return transcripts. However, all interview transcripts were included in the final analysis. To increase reliability and reduce personal interpretation of data, 25% of the transcripts were additionally coded by a second coder [56]. Any differences that arose due to dissimilar coding ideas or themes between coders were resolved through discussion between the coders [47].

## 3. Results

Semi-structured interviews provided insight into the beliefs and perceived factors influencing participants’ consumption of discretionary foods. Major themes that consistently arose from the participants’ responses were: (1) psychological factors (2) intrinsic qualities of EDNP foods, (3) social factors, (4) accessibility and affordability and (5) health related beliefs. The key findings reported within each of these themes are discussed in the following section. Differences between living arrangement groups were identified within theme 5.

### 3.1. Theme 1: Psychological Factors

Theme 1 includes five psychological factors (sub-themes) that participants thought influenced their consumption of EDNP foods. These included motivation, perceived behaviour control (habits and self-restraint), impulsivity, craving and coping strategies. These psychological factors were commonly noted among all living arrangement groups.

#### 3.1.1. Motivation

Participants reported that motivation was an important factor that determined their compulsion to make unhealthy food choices. Several factors contributed towards motivation. Two participants mentioned that a strong positive motivation to reduce their unhealthy food choices (and/or choosing healthy foods) was linked to getting older and changes in priorities that come with age. This was due to a desire to lay a foundation towards healthy food choices in early adulthood that can carry forward to future years. Other participants reported their positive motivations stemming from reading about food and developing an awareness about the impact of poor food choices on health. Making healthy food choices over unhealthy food choices was itself a strong motivator towards making improved food choices. This was understood as a means by which they took care of themselves and prioritised their health above the sensory attractions of unhealthy food choices.

*“I think personal motivation to become healthier, and I really wanted to set up healthy habits in my early 20s for the remainder of my adulthood.”*

Participant 37

*“I’m quite interested in looking through cookbooks and reading about food. I’m sure there’s a lot of people that wouldn’t have any interest in doing that. Being motivated to eat well, I also think there’d be a lot of people that wouldn’t necessarily care about what they eat so much as long as it tastes good and fills them up.”*

Participant 18

In contrast, a perceived lack of time and a busy life contributed towards decreased motivation for consuming healthy foods.

*“When I was working full time, if you are getting home late, you can be less motivated for example, to cook yourself a fresh healthy dinner…. So, time would be another factor that might make me eat more unhealthy food.”*

Participant 34

#### 3.1.2. Habits

A sense of automaticity and uncontrollability was associated with the consumption of EDNP foods in the form of snacks. Increased access, coupled with a lack of cognitive reasoning, led to making poor food choices.

*“At home…sometimes we drink and get a bit hungry, and then unknowingly, the first thing you’ll gravitate towards is snacks.”*

Participant 3

*“I tend to snack more at home as opposed to when I’m out of the house. So, being at home most days, I do find myself snacking more often unknowingly.”*

Participant 31

Interestingly, three participants thought that reducing their access to EDNP foods was a useful strategy to reduce consumption of EDNP foods.

*“….Because we used to have access to them, I use to eat them before…. I reduced buying junk foods… I realise if I do not have anything in the house, then I cannot eat them and so I tend to eat better now.”*

Participant 35

Another participant suggested that changing the habit of making unhealthy food choices required the need for an emotional transformation. This could be negotiated by focussing on the long-term health costs associated with making short-term and convenient low-cost unhealthy food choices. Reducing unhealthy food choices for its own health benefit was a helpful strategy.

*“If you’re used to just buying unhealthy food and then you have to change your habit, then it’s not only a financial cost, although it might be cheaper… it’s an emotional cost, to change your habits in the long-term…. I have to fight against myself normally, just to not take the easy option, but I’m going to pay for it later…. on an emotional level they’re (healthy food) a little bit more expensive to prepare and buy fresh food, but I know in the long-term it’s far cheaper.”*

Participant 2

#### 3.1.3. Self-Restraint

Four participants mentioned the practise of self-restraint to regulate consumption of EDNP foods. However, they suggested that practising self-restraint by completely avoiding EDNP foods was associated with periods of binge eating. Therefore, instead of complete avoidance, occasional consumption of EDNP foods was perceived as more sustainable.

*“I think it’s very easy to fall into a trap of restricting yourself and then eating things that aren’t healthy because you’ve deprived your body in a way, if that makes sense… I think also it encourages you to eat more, so that’s bad.”*

Participant 24

The comments of two other participants proposed that exercising control over food consumption decreased quality of life.

*“Food’s such an integral part of my life that I don’t want to limit or restrict that. Because I do think as though that’s where my happiness is derived from and it directly relates to my quality of life.”*

Participant 21

Familial and peer-support were viewed as central to the ability to successfully overcome discretionary food consumption habits.

*“…But getting rid of all these things in your life, one needs to be really strict with your diet. And then family members… or the friends need to be really supportive.”*

Participant 9

#### 3.1.4. Impulsivity

Five participants reported that a lack of logical reasoning and spontaneous decisions led them to the consumption of EDNP foods. These participants expressed a carefree attitude and did not consider the impact of making unhealthy food choices.

*“If I see someone have unhealthy food or talk about unhealthy food, I might want it…. If they’re talking about yogurt, I would immediately want yogurt as well. It’s not about healthy or unhealthy.”*

Participant 22

*“I feel that I don’t want to care about anything whether it (unhealthy food) will do any good for my body. I just need something to eat.”*

Participant 28

In contrast, one participant used planning as a strategy to avoid impulsive food purchases.

*“I’m not particularly drawn to things once I’m in the shop because I often have an idea about what I want to cook or what we’re planning to make throughout the week…. I probably would go in with a bit of a plan.”*

Participant 17

#### 3.1.5. Craving

Eleven participants associated EDNP foods with a sense of immediate gratification. Further, the urge for consumption of EDNP foods stemmed from an intense desire for tasty and spicy foods. EDNP foods being convenient were thus preferred over healthy food choices at that point in time. Further, these interviewees thought it was important for them to satisfy their food cravings to avoid their continuance.

*“When I’m craving something, let’s just use that as an example. When I’m really craving halal snack pack or HSP, it’s because I really crave the taste of it. I want something salty and savoury, and specifically it’s just a craving.”*

Participant 29

*“I will generally eat it because if I don’t, I’m just going to keep wanting it.”*

Participant 18

When participants discussed the satiation capacity of foods, they were often described through dichotomies positioning EDNP foods negatively in opposition to healthy foods. In these dualisms, EDNP foods were associated with decreased satiation capacity and ability to fulfil nutrient requirements. Despite this knowledge, casting EDNP foods as a way of satisfying one’s food cravings seemed to grant participants permission to consume EDNP foods.

*“I mean not full, not feeling very satiated but I also feel my cravings are satisfied.”*

Participant 36

*“Unhealthy foods often are not very nutrient dense… you’re not getting what your body needs… organ health and sustenance and being able to be alert and mentally well and being able to feel your best self.”*

Participant 21

#### 3.1.6. Coping Strategies

Seven participants thought that the consumption of EDNP foods was a way of seeking comfort during periods of emotional instability. These periods included those of feeling depressed or upset.

*“I definitely think that it’s tied to your emotions. If I’m a bit sad or tired, I’m a lot more likely to eat unhealthy foods. I want to eat more chocolate. I want to get takeaway.”*

Participant 24

*“I’m cranky or if I just feel like I need something really tasty to cheer myself up then I’m more likely to have unhealthy food. I want something that will make me feel better and it’s usually the instant gratification that you get from eating unhealthy foods.”*

Participant 16

Six other participants reported that consumption of EDNP foods was sought as a comfort mechanism to deal with periods of increased stress such as busy schedules including working towards a deadline. Being stressed contributed towards being less concerned about making EDNP food choices.

*“If I was stressed, I think I’d have more snacks… eat a lot more biscuits So, it’s normally if I have like a deadline or something to work towards.”*

Participant 35

*“Then another thing I found out is once I get back from work, I think that’s how I release my stress. I feel more relaxed (eating unhealthy food).”*

Participant 9

*“I don’t really eat unhealthy food unless I’m stressed…like Fish fingers or chips.”*

Participant 26

### 3.2. Theme 2: Intrinsic Qualities of EDNP Foods

The intrinsic qualities of EDNP foods, such as taste, appeal and convenience were identified as important and reinforcing factors that influenced consumption. Eight participants described the consumption of EDNP foods as a treat to reward themselves. This was more apparent among the male participants. EDNP foods were most frequently referred to as ‘treat’ foods and brought about immediate gratification.

*“It’s always the taste and flavour…. Or maybe the presentation of the food that affects purchase of unhealthy food.”*

Participant 11

*“I will often think of it as something like a treat. I haven’t had a burger from McDonald’s in ages and I’ve had this now, and “Oh, gosh, it tastes so good”.*

Participant 22

### 3.3. Theme 3: Social Factors

#### 3.3.1. Socialization

It appears that it was a perceived normative practice to consume EDNP foods in social (peer) and familial gatherings. As such, two participants reported that the consumption of EDNP foods were seen as a medium of improving social bonding. Consumption of EDNP foods over healthy foods with peers in social situations symbolised one’s flexibility of food choices which portrayed a carefree attitude. This in turn led to enjoyment, relaxation, cohesion and acceptability among peers. Consumption of healthy foods in these situations was considered either as a socially unacceptable alternative or less appealing than EDNP foods.

*“… Being able to be a little bit flexible with what you eat especially in terms of socializing with family or friends…unhealthy food brings people together… that’s nice thing to be able to do.”*

Participant 33

*“I’m more likely to eat it (unhealthy food) if other people are doing it. I think it can be a socially cohesive thing, like we eat things together and it’s almost a bonding experience.”*

Participant 8

Consumption of EDNP foods was seen to be a social ritual, being a central part of special occasions such as familial celebrations as well as peer gatherings.

*“I think it’s maybe the novelty of hanging out with friends when it’s a special occasion…. we feel like we could eat a little bit more.”*

Participant 12

*“I think we people think that that is the only way to celebrate. Family celebration… that is only foods especially unhealthy… that is the only source of celebration we have.”*

Participant 9

#### 3.3.2. Social Norms and Social Pressure

Familial influence and household food practices along with living arrangements were perceived to have a strong influence on attitudes and behaviours relating to the consumption of EDNP foods at home. Most participants noted reduced or little encouragement from families towards the consumption of EDNP foods. In contrast, more often EDNP foods were consumed when with peers. Social norms and social pressures to conform to food choices made by peers played an important role in determining young adults’ consumption of EDNP foods.

*“My whole family is strict. It’s probably my friends, I’m encouraged to eat…. it’s just like I feel it’s easier to eat unhealthy foods when I’m with my friends.”*

Participant 4

*“Probably friends, if they like unhealthy food or if you go out to eat somewhere and they’re all ordering unhealthy food, then that’s an approval for everyone to do the same thing… whereas with family you’d be more exposed to traditional foods, and they always eat at home.”*

Participant 16

*“Family never. They ask me to eat healthy food. They asked me to avoid unhealthy junk foods but my friends like junk food, so I must give company to them.”*

Participant 19

In contrast, four young adults did not perceive any social pressure to conform to unhealthy food choices and reported that their social environment did not influence their consumption of EDNP foods.

*“I don’t feel that there would be anyone that would influence me. I think most people I speak to are quite reasonable. And they are probably of the mindset that having unhealthy foods in moderation isn’t an issue.”*

Participant 37

Even in everyday situations where young adults consume food such as during lunches in schools or in universities, the consumption of foods like those eaten by others, was seen to be an important part of socialising with friends. These similar foods were most often identified as EDNP foods. Three participants expressed fear about consuming healthy foods, (or being very rigid about their food choices), as it could potentially lead to a lack of acceptance among the peer group and hence social isolation.

*“There could be an advantage of being able to fit in. There could be a group that ate only unhealthy food. Therefore, it could be a factor of fitting into a certain peer group. Because they might see you as being, too different if you’re the one that’s out there eating a chicken salad.”*

Participant 23

*“I think it becomes very hard to enjoy things when you’re very strict on your diet or when you’re trying to only eat healthy. That’s why I think it’s not a good thing to be healthy all the time, because I think it can be a bit isolating.”*

Participant 24

#### 3.3.3. Social Media

Social media played an important role in the participants’ consumption of EDNP foods. Ten participants discussed promotions, advertisements, and their past experiences of encouragement towards consumption of EDNP foods whereas three others noted the consistent portrayal of misinformation in the media and the use of targeted advertisements.

*“You can have a celebrity advertising it. And so, people who see that advertisement, think, “That celebrity does this or uses this product. We need to get it so we can be like them.” That’s a bit of a negative connotation.”*

Participant 23

*“I think if I see an influencer or see something on social media and it might be a new snack, I’m definitely going to look at it when I’m in the shops…so that’s definitely something that affects my consumption.”*

Participant 21

### 3.4. Theme 4: Accessibility and Affordability

#### 3.4.1. Accessibility

Food environments played an important role in determining accessibility to foods, at home, social settings and times of the day. That is, increased access at home contributed towards consumption. Most social interactions occurred in places that served EDNP foods. Many fast-food outlets that served EDNP foods were accessed during the night as they were the only food outlets accessible then.

*“Even though you want to get more solid kind of food, at night most restaurants are probably closed, so, more often than not, the restaurants that are open are kebab shops and everything, and then those aren’t the most healthy choices… It’s just a lack of options mainly.”*

Participant 3

*“I think availability’s probably the biggest one. I wouldn’t eat a lot of chocolate if it wasn’t there. I wouldn’t go and buy it for myself. I’d only eat it if it’s here.”*

Participant 8

#### 3.4.2. Affordability

Discretionary foods were described as more cost-effective than healthy foods; preparation of food was associated with increased cost and efforts. In contrast, however, some participants also believed that it was cheaper to prepare their own food rather than purchasing discretionary foods.

*“Broadly, I think the advantages… the only one that I can think of is increasing access to cheaper foods. I don’t think there are many advantages to fast foods such as McDonald’s.”*

Participant 37

*“And it’s also a lot cheaper, I guess, to make your own food rather than to buy out (fast food), so I’d also like to minimize the cost of eating out.”*

Participant 16

### 3.5. Theme 5: Health Related Beliefs

The participants were aware of the many potential health consequences of consuming EDNP foods. Their health-related beliefs appeared to have some influence on their attitudes towards food consumption. Major differences between the three different living arrangement groups were noted in this theme. Two participants from independent households and two from parental households reported health-related concerns. Members of the shared households did not report any health-related concerns. 

These four participants (two each from independent and parental households) associated high consumption of EDNP foods with weight gain, diabetes, high blood pressure, high cholesterol levels, heart disease and respiratory diseases. Diabetes was the most frequently discussed health outcome and was usually related to a family member having had the condition.

*“But I don’t eat them just because I want to avoid all those diseases… because in my family, diabetes runs on my dad’s side, also high blood pressure and high cholesterol levels.”*

Participant 30

*“I think I do get a bit guilty when I eat unhealthy foods or foods that I perceive unhealthy, because I get worried about the impact of weight gain.”*

Participant 12

*“If you have it (unhealthy foods) on a regular basis, you could be cutting out a lot of your nutritional needs that help you have energy and thrive as a person.”*

Participant 32

Overall, five out of six components of the FRLM were mentioned by participants, these included concrete attributes, quality expectations, usage situations, cooking scripts and perceived consequences. Participants did not report on any aspects relating to the shopping scripts component of the FRLM. Further, while the FRLM does incorporate some aspects of impulsivity, it does not consider five other psychological factors identified in this study. These include motivation, restraint, cravings, coping strategies and habits. 

## 4. Discussion

The semi-structured interviews provided rich information concerning factors influencing participants’ consumption of EDNP foods. Five broad themes emerged. First, six psychological factors were identified which included motivation, habits, self-restraint, impulsivity, craving and coping strategies. The second theme listed intrinsic qualities of EDNP foods such as taste, appeal and convenience. The third theme, social factors, identified social norms, peer pressure and social media as key factors. Under the fourth theme, accessibility and affordability were noted as environmental factors. Lastly, theme five reported on health-related beliefs. Previous studies have noted motivation and dietary restraint as enablers of healthy eating among young adults [26]. However, taste, appeal, convenience, social norms, social media, accessibility, affordability, impulsivity, coping strategies and health-related beliefs have been the barriers to healthy eating among this age cohort [26]. Factors such as cravings, habits and peer pressure are novel, not having been previously identified as factors influencing young Australian adults’ food consumption. 

### 4.1. Theme 1: Six Psychological Factors

While the FRLM is an important model suggesting a framework for studying food consumption behaviours [57], our findings suggest that the FRLM does not consider motivation, restraint, cravings, coping strategies and habits reported by the participants as psychological factors driving their consumption of EDNP foods. 

Firstly, participants reported motivation as a key factor influencing their affinity towards EDNP foods. Motivation has been noted as a missing component of FRLM in the past [35]. The category and quality of motivation (autonomous or controlled motivation) is more important than the total amount of motivation [58]. Autonomous motivation refers to engaging in an activity because it is seen to be congruent with intrinsic objectives or outcomes and emanates from the self and it has been identified as an enabler of healthy eating [26]. Contrastingly, controlled motivation (the expectation of rewards or avoiding punishment for an activity which fosters worthiness, self-esteem, avoidance of guilt and shame [58]) is associated with a focus on short-term benefits and decreased adherence to healthy eating [58]. Five participants reported examples of controlled motivation, seeking rewards in the form of unhealthy treats for hard work. In contrast, three participants discussed autonomous motivation where reading or gaining knowledge about healthy foods, preparing and consuming healthy foods were gratifying which in turn enabled them to decrease their consumption of discretionary foods. Therefore, it appears that controlled motivation may be playing a role in negotiating consumption of discretionary foods among some participants. However, due to the qualitative nature of the present study, it is not possible to draw any causal relationships between discretionary food intakes and the two forms of motivation. Future quantitative studies are required to clarify these relationships. If the relationship between autonomous motivation and healthy eating is confirmed positively, then future health promotion could focus on enhancing healthy eating using autonomous motivators. This may in turn support positive psychological health and reduce the consumption of discretionary foods among young adults that are gratifying in the short-term [26].

Secondly, some of the participants’ reported links between stress and their consumption of discretionary foods. This is supported by previous research among young adults [26]. However, these reports were made by only six participants which suggests that other factors may be influencing consumption of discretionary foods.

Thirdly, some participants reported flexible restraint but not rigid restraint as a helpful strategy for them to avoid excessive consumption of EDNP foods. Dietary restraint refers to efforts to limit food intake in order to lose or maintain weight [59]. Again, this is supported by past research [59]. Some types of restraint may be more effective than others in preventing excessive eating [59]. For example, flexible restraint may prevent excessive intake of palatable non-nutritious foods [59]. In contrast, rigid restraint may be ineffective in regulating intake of discretionary foods [59]. However, it is less possible to draw any inferences due to the qualitative nature of the study.

Fourth, habit strength may be an important predictor of unhealthy snacking behaviour among adults [36]. Several participants in the present study reported healthy eating habits were important for them. Participants suggested focussing on long-term negative implications of discretionary foods as a helpful strategy for improving discretionary eating habits. Additionally, focussing on the health benefits of avoiding discretionary food habits were suggested. This underscores the need for future studies to examine the role of habit strength of discretionary food consumption among young adults. 

Fifth, several of our participants appeared to be impulsive consumers of discretionary foods. The FRLM takes into account some aspects of impulsivity as part of the usage situations component. Impulsivity is a multidimensional construct characterised by an inclination towards taking excessive risks, unplanned and rapid actions that are ill-considered and inadequate in a given situation and is frequently linked to an inability to delay gratification [60]. Our findings are supported by a Polish study of young adults, which showed a strong relationship between impulsivity and unhealthy food choices [40]. Consumer segmentation studies have noted that impulsively involved Australian adults (aged 19 years and above) reported higher consumption of fast foods, convenience foods and unhealthy snacks than the ‘rational, health conscious’ and ‘uninvolved consumers’ [35]. However, past studies have not explored the association between impulsivity and gratification exclusively focussing on young Australian adults’ EDNP food behaviours. This lack of research provides an opportunity for further exploration. 

Finally, several participants saw discretionary foods as rewards (treats) for hard work. There is a need to address this perception. A health promotion campaign targeting this age cohort is required, which would highlight the high energy and poor nutrient density of discretionary foods, encourage flexible restraint and moderation whilst promoting alternative healthy treats and rewards. 

Therefore, the present study has shown that some participants reported associations between motivation, habits, coping strategies, restraint, impulsivity, and discretionary food intakes. In each case, we recommend that future quantitative studies be conducted to test these associations.

### 4.2. Theme 2: Intrinsic Qualities of EDNP Foods

The factors identified under this theme include taste, convenience and appeal. These findings are in line with past research. A study by Hebden et al. (2015) concluded that taste (hedonism) was the most influential factor that determined food selection among young adults [25]. This was followed by convenience (availability) and cost; however, health and nutritional value was viewed as being least important among 18–24-year old Australians [25]. A study by Hayley et al. (2015) demonstrated that personal values such as universalism, power and security was associated with meat eating preferences among all Australian adults [61]. Our study contributes further towards this understanding by observing that male participants tended to exhibit greater preference towards taste, appeal, and convenience. That is, their desire for seeking immediate gratification from discretionary foods may signify that male participants could be increasingly hedonistic. However, there is a lack of quantitative evidence to comment on which of the intrinsic factors relate to EDNP food behaviours among current study participants. This demonstrates the need for further examination. The FRLM considers the intrinsic factors identified in this study as part of the quality aspects and the concrete attributes components. The factor of convenience is included as part of the cooking scripts component of the FRLM.

### 4.3. Theme 3: Social Factors

Discretionary food and beverage consumption seems to be an important part of participants’ social contexts. For example, it appears to be a social norm to consume discretionary foods during social activities where exchange or consumption of discretionary foods was an additional way of portraying social bonding and flexibility. Social norms, social isolation and social pressures are responsible for conforming to food choices made by peers and played an important role in negotiating participant’s consumption of EDNP foods. Social media for example advertisements, including celebrity endorsements, promotions such as appealing images, novel foods and discounts encouraged participants’ consumption of discretionary foods. The FRLM takes into consideration the social factors via the usage situation component.

Peer social norms play a significant role in young adults’ food intake including those of discretionary foods [62]. Our study contributes further to this understanding by noting that pressure to conform and the fear of social isolation played a key role for the adherence to social norms among study participants. The need for identity exploration and desire for establishing social connections including belongingness associated with the transitional period of young adulthood [14] could be potential reasons for adherence to social norms among current study participants. Higgs et al. (2015) suggest that norm following is most likely when there is a lack of clarity concerning what represents suitable behaviour and when there is a great sense of shared identity with the norm referent group [41]. Further, a lack of experience and lack of knowledge concerning what constitutes healthy eating could also be important reasons for discretionary food consumption. This may be consistent with their reported attention to social media including promotions that encouraged and influenced young adults’ consumption of discretionary foods. 

Because of the power of peer influence, health-promoting social marketing strategies could aim to tap into informal social networks [24] to promote healthy eating norms which are informative, engaging, and convincing. Given the increased use of social media observed among young adults [63], and further confirmed by current study participants, this may be a promising avenue. It is also important to increase the social desirability of healthier alternatives, particularly in social gatherings. For example, this might be done by fostering healthy eating on social media by portraying friends eating healthily at social events or reporting on healthy eating trends targeting this age cohort [26]. This may in turn have the potential to influence the high EDNP food intake noted amongst young adults.

### 4.4. Theme 4: Accessibility and Affordability

Several participants mentioned food accessibility and affordability as important determinants of their unhealthy food choices. That is, participants perceived easy access to discretionary foods at home, late nights and in places of social interactions such as restaurants and pubs as factors encouraging consumption of discretionary foods. They further believed that unhealthy food choices were convenient and saved time and effort. The FRLM takes into heed affordability through the concrete attributes component and that of accessibility through the usage situations component. 

The relative ease of access to discretionary foods (explained by price, location, time, and effort saving) has been a commonly reported as a barrier to healthy eating among young adults [26]. Positive associations between increased proximity to food outlets such as the fast-food outlets and body mass index have been noted before [64]. Participants believed that limited access to healthy foods during late hours at night was an important factor that significantly influenced their consumption of discretionary foods during the weekends. These reflect the need for improving access to healthy foods beyond usual work hours especially during the weekends. 

The common perception that an unhealthy diet is cheaper than a healthier one is exacerbated by the financial instability of young adults [26]. Past studies have noted the greater cost of healthier diets in the United Kingdom [65], United States of America [66] and in Australia [67,68], though the difference in affordability may be less than some studies claim [69]. Nevertheless, convenient, less expensive alternatives such as fast foods are often preferred by many young adults [70,71]. Affordability relates to ‘price’ and the perception that discretionary foods are cheaper strongly influences the food choices of young adults [69].

These results further support past findings which suggest that young adults’ purchases of discretionary foods are largely determined by increased cost and limited access to healthy foods [72]. It is therefore important for further public health interventions and policy to address accessibility and affordability of discretionary foods. 

### 4.5. Theme 5: Health Related Beliefs

The health-related beliefs identified in this study closely relate to the perceived consequence component of the FRLM. The FRLM considers the perceived consequences of consumption of food and its significance through the perceived consequence component. 

Only four participants in our study belonging to independent (n = 2) and parental households (n = 2) expressed concerns about health consequences associated with consumption of discretionary foods. This agrees with previous studies that have reported fewer concerns regarding the health consequences of food choices among young adults [73]. Further, our study participants’ health concerns seemed to have risen from personal experience that is through an acquaintance such as a friend or a family members’ association with chronic disease. 

Subsequently, these findings suggest the need for tailoring health communication messages to highlight young adults’ vulnerability to negative health consequences of making unhealthy food choices. It is important for these messages to employ positive messages and focus on short-term reinforcements in combination with leisure activities and fun [74,75]. 

Overall, the FRLM takes into consideration some of the factors noted in this study as influencers of discretionary food consumption. The present findings report five of the six components of the FRLM (concrete attributes, quality expectations, usage situations, cooking scripts and perceived consequences). However, the participants did not discuss aspects relating to the shopping scripts. This could be due to the nature of the questions asked that in they may have focussed less on eliciting responses relating to food shopping behaviour. More importantly, the FRLM omits important psychological factors such as motivation, restraint, cravings, coping strategies and habits identified by participants as their influencers over discretionary food intakes. Our findings are in support of the criticisms of the FRLM noted in past research concerning the lack of consideration for psychological aspects such as motivation and habits [35,36]. Our findings suggest that the FRLM may need to be extended to accommodate additional factors in its application to the unhealthy food choices of this age cohort. However, it is not possible to draw any firm conclusions due to the qualitative nature of the study and future quantitative research could confirm these findings. A new conceptual model has been suggested with socio-psychological factors such as motivation, habits, restraint, coping strategies and impulsivity added to the FRLM [29] and this model requires further testing. 

## 5. Strengths and Limitations

Until now, most studies of young adults’ food perceptions have focussed on perceptions of healthy eating and factors influencing the consumption of healthy foods. However, this is one of the first studies that reports influences over the consumption of EDNP foods. The findings from this exploratory study are particularly valuable in the light of limited research regarding young adults’ discretionary food behaviours. The use of qualitative methods such as interview questions and follow-up probes helped to provide in-depth insights by identifying a range of factors influencing discretionary food consumption. Additionally, the use of hierarchical coding using the template analysis technique helped to capture rich and detailed aspects of the data. These novel findings inform future quantitative research by highlighting likely influences over discretionary food intakes.

Whilst the present study provides novel insights, there are some limitations. Firstly, the qualitative nature of the study limits the possibility of generalizing the study’s findings to the population at large. However, findings identified in this formative study could form a basis for further quantitative examinations. Secondly, because of logistic limitations, it was not possible to measure participants’ dietary intake and to draw any causal relationships with the identified factors. 

## 6. Conclusions

Psychological factors, social factors, accessibility and affordability, intrinsic qualities of EDNP foods and health related beliefs were noted as major themes influencing the reported consumption of EDNP foods. The study investigated a previously under-researched topic, and its findings have important implications for research and healthy eating practices. Social marketing campaigns that promote healthy eating norms whilst addressing social and environmental factors influencing consumption of EDNP foods could be suggested. An awareness campaign that highlights health consequences of increased consumption of EDNP foods could be beneficial. There is a need to increase the social desirability of healthier alternatives, particularly in social gatherings of young adults. Further quantitative research is required to examine the accessibility, affordability, social, and psychological influences over young Australian adults’ consumption of EDNP foods. The FRLM may need to be extended to accommodate additional factors in its application to the unhealthy food choices of young Australian adults.

## Figures and Tables

**Table 1:** Interview questions.

1	Main Question	How do you feel about consuming unhealthy foods?
2	Main Question	Who encourages you to consume unhealthy foods?
3	Main Question	Who are the ones who would approve your consumption of unhealthy foods?
	Probe	How about friends, family?
4	Main Question	Who are the ones who would disapprove your consumption of unhealthy foods?
	Probe	How about friends, family?
5	Main Question	When and with whom do you normally consume unhealthy foods?
	Probe	Do you eat different foods when you are with your friends or family or when on your own?
6	Main question	What are the factors that would contribute towards your consumption of unhealthy foods?
	Probe	What are the factors that would make it difficult for you to consume unhealthy foods?
	Probe	What are the factors that would make it easy for you to consume unhealthy foods?
	Probe	How do social media/advertisements influence your consumption of unhealthy foods?

## Data Availability

Data were newly collected for the purpose of the study. However, data cannot be made available publicly as it is subject to ethics as outlined by the Deakin University Faculty of Health Ethics Advisory Group.
